# Performance Analysis of IRS-Assisted THz Communication Systems over *α*-*μ* Fading Channels with Pointing Errors

**DOI:** 10.3390/s23167028

**Published:** 2023-08-08

**Authors:** Sajid Hussain Alvi, Bakhtiar Ali, Jawad Mirza, Muhammad Awais Javed, Adnan Fida, Byung Moo Lee, Tariq Bashir

**Affiliations:** 1 Department of Physics, COMSATS University, Islamabad 45550, Pakistan; sajid_hussain@comsats.edu.pk; 2 Department of Electrical and Computer Engineering, COMSATS University, Islamabad 45550, Pakistan; bakhtiar_ali@comsats.edu.pk (B.A.); jaydee.mirza@gmail.com (J.M.); awais.javed@comsats.edu.pk (M.A.J.); adnan_fida@comsats.edu.pk (A.F.); tariq_basir@comsats.edu.pk (T.B.); 3 Department of Intelligent Mechatronics Engineering, and Convergence Engineering for Intelligent Drone, Sejong University, Seoul 05006, Republic of Korea

**Keywords:** intelligent reflecting surface, terahertz, *α*-*μ* fading, ergodic capacity, outage probability

## Abstract

In this paper, we analyze the performance of an intelligent reflecting surface (IRS)-aided terahertz (THz) wireless communication system with pointing errors. Specifically, we derive closed-form analytical expressions for the upper bounded ergodic capacity and approximate expression of the outage probability. We adopt an α-μ fading channel model for our analysis that is experimentally demonstrated to be a good fit for THz small-scale fading statistics, especially in indoor communication scenarios. In the proposed analysis, the statistical distribution of the α-μ fading channel is used to derive analytical expressions for the ergodic capacity and outage probability. Our proposed analysis considers not only the IRS reflected channels, but also the direct channel between the communication nodes. The results of the derived analytical expressions are validated through Monte Carlo simulations. Through simulations, it has been noticed that pointing errors degrade the performance of the IRS-assisted THz wireless communication system which can be compensated by deploying an IRS having a large number of reflecting elements.

## 1. Introduction

It is expected that 6G networks will achieve exceptionally higher performance in terms of data rate, latency, connectivity, reliability and security compared to presently available 5G networks [1]. For this purpose, several enabling technologies have been identified in the recent literature for 6G systems. One such promising technology is the intelligent reflecting surface (IRS), which can enable a smart and programmable wireless propagation environment [2]. In general, an IRS is made up of a 2D metasurface which consists of a large number of passive reflective elements. These elements induce a phase shift on the incident signal and reflect it accordingly, i.e., either to improve the signal strength of users or null out specific users [3].

Numerous studies have statistically characterized various performance metrics in THz wireless communication systems. As the THz wireless link depends heavily on the line-of-sight (LoS) component of the received signal, the channel modeling was mostly dominated by large-scale fading phenomena such as deterministic path loss and shadowing [4]. However, recent measurement campaigns have also observed small-scale fading phenomenon in THz links due to the presence of non-line-of-sight (NLoS) links.

The majority of the existing work on the performance analysis of IRS-assisted single-input single-output (SISO) links have modeled small-scale fading statistics using Rayleigh [5], Rician [6] and Nakagami-*m* [7] distributions. For THz communications, it has recently been demonstrated through measurements in different scenarios that the α-μ fading model yields a good fit to THz small-scale fading statistics, which is justified by the Kolmogorov–Smirnov test [4]. In addition to the α-μ fading model, the fluctuating two-ray (FTR) model has also been shown to accurately model the small-scale fading statistics of THz links. However, for the modeling of indoor THz communication scenarios, the α-μ fading model has been shown to be more precise [4]. Furthermore, unlike FTR model distribution, the α-μ fading distribution is analytically tractable. There are few studies [8,9] which have carried out a performance analysis of a THz communication system over α-μ fading channels. Moreover, the work on the performance characterization of IRS-assisted THz wireless links over α-μ fading channels is far more scarce. For instance, in [10], the authors derive expressions for the outage probability, ergodic capacity and average bit-error-rate (BER) for the IRS-assisted THz system. The performance analysis carried out in [10] considers IRS-assisted links where the direct link is absent.

In this paper, we present a performance analysis of an IRS-assisted THz wireless communication system over α-μ fading channels. Unlike [10], we consider both IRS-assisted and direct links in our analysis and derive closed-form upper bounded ergodic capacity and outage probability expressions. It is important to mention that by considering the direct path, obtaining the exact analysis becomes a non-trivial task due to the coupling between the channels of direct and IRS-assisted links. The expression derived for the outage probability is expressed in terms of Fox’s H-function. The proposed ergodic capacity expression provides a tight upper bound with moderate and large numbers of IRS reflective elements.

## 2. System and Channel Models

**Figure 1:**
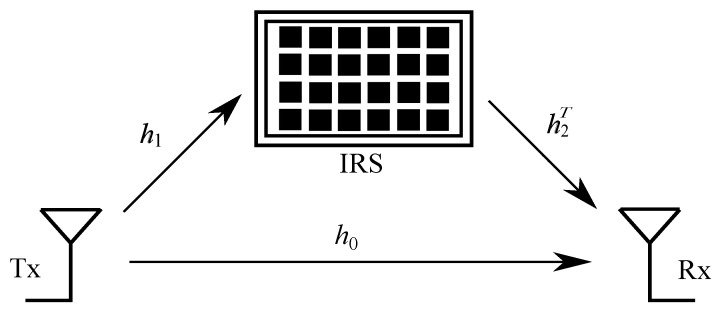
An illustration of the IRS-assisted SISO communication system.

Consider an IRS-aided wireless communication system consisting of a transmitter (Tx), receiver (Rx) and IRS, as shown in Figure 1. It is assumed that the Tx and Rx are equipped with a single antenna each. The total number of reflecting elements in the IRS is represented by *N*. Let $d_{i}$, *i*∈$\left\{0,1,2\right\}$ represent the distances of the Tx–Rx, Tx–IRS and IRS–Rx links, respectively. The Rx receives the signals from the Tx–Rx and Tx–IRS–Rx links, and therefore the received signal at the Rx can be written as

(1) $$\begin{array}{c} r_{1}=\sqrt{P}H_{1}H_{2}\left(h_{2}^{T}\Phi h_{1}\right)s+\sqrt{P}H_{0}h_{0}s+n, \end{array}$$

The received signal at the Rx in case of pointing errors can be written as

(2) $$\begin{array}{c} r_{2}=\sqrt{P}H_{1}H_{2}\left(h_{p2}^{T}\Theta h_{p1}\right)s+\sqrt{P}H_{0}h_{p0}s+n, \end{array}$$

where *P* denotes the total transmit power at the Tx, *s* represents the transmitted signal with unit energy and *n* is a zero mean additive white Gaussian noise (AWGN) at the Rx with the power spectral density denoted by $N_{0}$ (Watts/Hz). The deterministic path gain coefficient $H_{i}$ captures the effect of the distance, antenna gains, carrier frequency and molecular absorption coefficient for the *i*th link. Meanwhile, the variables $h_{pi}$ and $h_{i}$ model the small-scale fading for the *i*th link with and without pointing error, respectively, according to the α-μ fading distribution. It may be noted that $h_{pj}$ and $h_{j}$, where *j*∈$\left\{1,2\right\}$, are N×1 channel vectors with and without pointing errors, respectively. For example, $h_{1}$ and $h_{2}$ denote the channels of the Tx–IRS and IRS–Rx links, respectively, without pointing errors. The phase-shift matrix of size N×N is given by $\Phi ≜diag\left\{\phi \right\}$ with $\phi ={\left[e^{j\phi_{1}},e^{j\phi_{2}},\ldots ,e^{j\phi_{N}}\right]}^{T}$, where $\phi_{n}\in \left[0,2\pi \right)$ represents the phase of the *n*th element in the IRS.

The phase shifts at the IRS are controlled by the Tx through a wired/wireless link between the Tx and IRS microcontroller. There are few studies that exploit dual-polarized IRS design [11,12]; however, in this work we assume a single-polarized IRS. Furthermore, assuming perfect channel state information (CSI) at the Tx and considering the optimal phase-shift matrix Φ [6], the maximum SNR of the system can be expressed as

(3) $$\begin{array}{cc} \gamma_{max} & =\frac{P|H_{1}{|}^{2}{\left|H_{2}\right|}^{2}}{N_{0}}{\left(\sum_{n=1}^{N}|h_{2,n}\left|\right|h_{1,n}\left|+|H|\right|h_{0}|\right)}^{2}\\ \; & =\overset{0}{\gamma }{\left(\sum_{n=1}^{N}|h_{2,n}\left|\right|h_{1,n}\left|+H\right|h_{0}|\right)}^{2}, \end{array}$$

where $\overset{0}{\gamma }=\frac{P|H_{1}{|}^{2}{\left|H_{2}\right|}^{2}}{N_{0}}$ is the SNR and $H=\frac{|H_{0}|}{|H_{1}\left|\right|H_{2}|}$.

### 2.1. Deterministic Path Loss Model

The path gain coefficients for the Tx–Rx, Tx–IRS and IRS–Rx links can be written as [8,13]

(4) $$\begin{array}{c} H_{0}=\frac{c\sqrt{G_{t}G_{r}}}{4\pi f\;d_{0}}\;exp\left(-0.5k\left(f,T,\psi ,p\right)d_{0}\right), \end{array}$$

(5) $$\begin{array}{c} H_{1}=\frac{c\sqrt{G_{t}N^{2}}}{4\pi f\;d_{1}}\;exp\left(-0.5k\left(f,T,\psi ,p\right)d_{1}\right), \end{array}$$

(6) $$\begin{array}{c} H_{2}=\frac{c\sqrt{N^{2}G_{r}}}{4\pi f\;d_{2}}\;exp\left(-0.5k\left(f,T,\psi ,p\right)d_{2}\right), \end{array}$$

where *c* denotes the velocity of light and *f* is the carrier frequency. $G_{t}$ and $G_{r}$ represent the antenna gains at the Tx and Rx, respectively. The term $k\left(f,T,\psi ,p\right)$ is known as the molecular absorption coefficient, which depends on frequency *f*, temperature *T*, relative humidity ψ and atmospheric pressure *p*. This term can be expressed as [8]

(7) $$\begin{array}{cc} k\left(f,T,\psi ,p\right) & =\frac{q_{1}\;\nu \left(q_{2}\;\nu +q_{3}\right)}{{\left(q_{4}\;\nu +q_{5}\right)}^{2}+{\left(\frac{f}{100c}-p_{1}\right)}^{2}}\\ \; & +\frac{q_{6}\;\nu \left(q_{7}\;\nu +q_{8}\right)}{{\left(q_{9}\;\nu +q_{10}\right)}^{2}+{\left(\frac{f}{100c}-p_{2}\right)}^{2}}\\ \; & +c_{1}\;f^{3}+c_{2}\;f^{2}+c_{3}\;f+c_{4}, \end{array}$$

where $\nu =\frac{p_{w}\left(T,p\right)}{p}$ and $p_{w}\left(T,p\right)$ denotes the saturated water vapour partial pressure and can be evaluated based on Buck’s equation [8,13]. The model presented in (7) was shown to have great accuracy for up to one kilometer (Km) links in standard atmospheric conditions, i.e., T=296 $K^{\circ }$, p=101,325 Pa and ψ=0.5 [13]. The rest of the values in (7) can be found in ([8], Table 2). Then, $p_{w}\left(T,p\right)$ can be evaluated as [13]

(8) $$\begin{array}{c} p_{w}\left(T,p\right)=w_{1}\left(w_{2}+w_{3}\;p\right)\;exp\left(\frac{w_{4}\left(T-w_{5}\right)}{T-w_{6}}\right), \end{array}$$

where $w_{1}=6.1121$, $w_{2}=1.0007$, $w_{3}=3.46\times 10^{-8}$ 1/Pa, $w_{4}=17.502$ $K^{\circ }$, $w_{5}=273.15$ $K^{\circ }$ and $w_{6}=32.18$ $K^{\circ }$.

### 2.2. Fading Channel Model

Let $h_{i}$ model the α-μ distributed independent but not necessarily identically distributed (i.n.i.d) flat-fading for the *i*th links. Then, the PDF of $h_{i}$ is given as [14]

(9) $$\begin{array}{c} f_{h_{i}}\left(h_{i}\right)=\frac{\alpha_{i}\mu_{i}^{\mu_{i}}h_{i}^{\alpha_{i}\mu_{i}-1}}{{\hat{h_{i}}}^{\alpha_{i}\mu_{i}}\Gamma \left(\mu_{i}\right)}exp\left(-\mu_{i}\frac{h_{i}^{\alpha_{i}}}{{\hat{h_{i}}}^{\alpha_{i}}}\right),\;\;h_{i}\geq 0 \end{array}$$

where $\Gamma \left(\cdot \right)$ is the Gamma function [15] and $\hat{h_{i}}=\sqrt[{\alpha_{i}}]{E\{h_{i}^{\alpha_{i}}\}}$ is the $\alpha_{i}$-root mean value. It may be noted that ${\hat{h_{i}}}^{\alpha_{i}}=\mu_{i}{\left(\frac{\Omega_{i}}{\xi_{i}}\right)}^{\frac{\alpha_{i}}{2}}$ ensures $E\{|h_{i}{|}^{2}\}=\Omega_{i}$, wherein $\xi_{i}=\frac{\Gamma \left(\mu_{i}+\frac{2}{\alpha_{i}}\right)}{\Gamma \left(\mu_{i}\right)}$. Furthermore, $|h_{i}{|}^{\alpha_{i}}∼G\left(\mu_{i},\frac{{\hat{h_{i}}}^{\alpha_{i}}}{\mu_{i}}\right)$ is a Gamma distributed random variable, $E\{|h_{i}\left|\right\}=\frac{\Gamma \left(\mu_{i}+\frac{1}{\alpha_{i}}\right)}{\Gamma \left(\mu_{i}\right)}\sqrt{\frac{\Omega_{i}}{\xi_{i}}}$ and *VAR*$\left\{\right|h_{i}\left|\right\}=\Omega_{i}\left\{1-\frac{1}{\xi_{i}}{\left(\frac{\Gamma \left(\mu_{i}+\frac{1}{\alpha_{i}}\right)}{\Gamma \left(\mu_{i}\right)}\right)}^{2}\right\}$.

The CDF of $h_{i}$ [14], Equation (8) can be written using the identity [15] Equation (8.356.3) as

(10) $$\begin{array}{c} F_{h_{i}}\left(h_{i}\right)=1-\frac{1}{\Gamma \left(\mu_{i}\right)}\;\Gamma \left(\mu_{i},\frac{\mu_{i}}{{\hat{h_{i}}}^{\alpha_{i}}}h_{i}^{\alpha_{i}}\right), \end{array}$$

where $\Gamma \left(\cdot ,\cdot \right)$ is the upper incomplete gamma function [15], Equation (8.350.2).

Let $y_{i}={\left|h_{i}\right|}^{2}$ represent the gain of the fading channel for the *i*th link, then the PDF of $y_{i}$ can be written as

(11) $$\begin{array}{c} f_{y_{i}}\left(y_{i}\right)=\frac{\alpha_{i}{\left(\frac{\xi_{i}}{\Omega_{i}}\right)}^{\frac{\alpha_{i}\mu_{i}}{2}}}{2\Gamma \left(\mu_{i}\right)}y_{i}^{\frac{\alpha_{i}\mu_{i}}{2}-1}exp\left(-{\left(\frac{\xi_{i}y_{i}}{\Omega_{i}}\right)}^{\frac{\alpha_{i}}{2}}\right).\;\;y_{i}\geq 0 \end{array}$$

The PDF of the fading variable $h_{pi}$ caused by pointing errors at the three links can be written as [8] Equation (4)

(12) $$\begin{array}{c} f_{h_{pi}}\left(x\right)=\frac{\phi_{i}}{S_{i}^{\phi_{i}}}\;x^{\phi_{i}-1},\;0\leq x\leq S_{i}, \end{array}$$

where $S_{i}={\left|erf\left(\xi \right)\right|}^{2}$ represents the fraction of the collected power when Tx–Rx antennas are fully aligned. We have ξ = $\sqrt{\frac{\pi }{2}}\times \frac{r_{1}}{w_{d1}}$, where $r_{1}$ is the radius of the effective area of the Rx antenna reception and $w_{d1}$ is the radius of the transmission beam footprint at a distance $d_{1}$. Moreover, $\phi_{i}=\frac{w_{eq}^{2}}{4\sigma_{s}^{2}}$ is a squared ratio of the equivalent beam radius at the Rx and the variance of pointing error displacement at the Rx, where $w_{eq}^{2}$ = $\frac{\sqrt{\pi }\;w_{d1}^{2}\;erf\left(\xi \right)}{2\;\xi \;exp\left(-\xi^{2}\right)}$ is the doubled jitter standard deviation [16]. The parameter values in (12) are set according to [10].

Let $|h_{pi}|$ = $|h_{p}h_{i}|$ model the combined effect of short-term and pointing error fading at the corresponding links, then its PDF can be written as [8] Equation (6)

(13) $$\begin{array}{c} f_{|h_{pi}|}\left(x\right)=\frac{\phi_{i}{\left(\mu_{i}\right)}^{\frac{\phi_{i}}{\alpha_{i}}}x^{\phi_{i}-1}}{{\left(S_{i}\;\hat{h_{i}}\right)}^{\phi_{i}}\Gamma \left(\mu_{i}\right)}\Gamma \left(\mu_{i}-\frac{\phi_{i}}{\alpha_{i}},\frac{\mu_{i}\;x^{\alpha_{i}}}{{\left(S_{i}\;\hat{h_{i}}\right)}^{\alpha_{i}}}\right), \end{array}$$

where $\Gamma \left(\cdot ,\cdot \right)$ is the upper incomplete gamma function [15], Equation (8.350.2) and $E\left\{|h_{pi}|\right\}$ = $\frac{\phi_{i}}{1+\phi_{i}}\times \frac{S_{i}\;\hat{h_{i}}}{\mu_{i}^{\frac{1}{\alpha_{i}}}}\times \frac{\Gamma \left(\mu_{i}+\frac{1}{\alpha_{i}}\right)}{\Gamma \left(\mu_{i}\right)}$. The CDF of $|h_{pi}|$ can be written as [16] Equation (6)

(14) $$\begin{array}{cc} F_{|h_{pi}|}\left(x\right) & =1-\frac{\phi_{i}\;x^{\phi_{i}}}{\alpha_{i}{\left(S_{i}\;\hat{h_{i}}\right)}^{\phi_{i}}}\sum_{k=0}^{\mu_{i}-1}\frac{{\left(\mu_{i}\right)}^{\frac{\phi_{i}}{\alpha_{i}}}}{k!}\Gamma \;\left(k-\frac{\phi_{i}}{\alpha_{i}},\frac{\mu_{i}\;x^{\alpha_{i}}}{{\left(S_{i}\;\hat{h_{i}}\right)}^{\alpha_{i}}}\right). \end{array}$$

Similarly, the PDF of $|h_{pi}{|}^{2}$ can be expressed as

(15) $$\begin{array}{c} f_{|h_{pi}{|}^{2}}\left(y\right)=\frac{\phi_{i}{\left(\mu_{i}\right)}^{\frac{\phi_{i}}{\alpha_{i}}}y^{\frac{\phi_{i}}{2}-1}}{2{\left(S_{i}\;\hat{h_{i}}\right)}^{\phi_{i}}\Gamma \left(\mu_{i}\right)}\Gamma \left(\mu_{i}-\frac{\phi_{i}}{\alpha_{i}},\frac{\mu_{i}\;y^{\frac{\alpha_{i}}{2}}}{{\left(S_{i}\;\hat{h_{i}}\right)}^{\alpha_{i}}}\right), \end{array}$$

with $E\left\{|h_{pi}{|}^{2}\right\}$ = $\xi_{i}\left(\frac{\phi_{i}}{2+\phi_{i}}\right){\left(\frac{S_{i}\;\hat{h_{i}}}{\mu_{i}^{\frac{1}{\alpha_{i}}}}\right)}^{2}$.

Using these preliminary results, the performance analysis of the studied system is presented in the following section.

## 3. Performance Analysis

The performance analysis of the proposed IRS-based THz system is presented in this section. In particular, closed-form expressions for the upper bounded ergodic capacity and approximated outage probability are derived in this section.

We assume that perfect channel state information (CSI) is available and therefore use the optimal phase-shift matrix Φ [6]. The maximum SNR of the system without pointing errors can be written using (1) as

(16) $$\begin{array}{cc} \gamma_{max1} & =\frac{P|H_{1}{|}^{2}{\left|H_{2}\right|}^{2}}{N_{0}}{\left(\sum_{n=1}^{N}|h_{2,n}\left|\right|h_{1,n}|+\frac{|H_{0}|}{|H_{1}\left|\right|H_{2}|}\left|h_{0}\right|\right)}^{2}\\ \; & =\overset{0}{\gamma }{\left(\sum_{n=1}^{N}|h_{2,n}\left|\right|h_{1,n}\left|+H\right|h_{0}|\right)}^{2}, \end{array}$$

Similarly, the maximum SNR of the system considering pointing errors can be written using (2) as

(17) $$\begin{array}{c} \gamma_{max2}=\overset{0}{\gamma }{\left(\sum_{i=1}^{N}|h_{p2,i}\left|\right|h_{p1,i}\left|+H\right|h_{p0}|\right)}^{2}, \end{array}$$

where *H* = $\frac{|H_{0}|}{|H_{1}\left|\right|H_{2}|}$ and $\overset{0}{\gamma }$ = $\frac{P|H_{1}{|}^{2}{\left|H_{2}\right|}^{2}}{N_{0}}$ is the SNR.

### 3.1. Capacity Analysis without Pointing Errors

A general equation for the ergodic capacity of a fading channel can be written as

(18) $$\begin{array}{c} \bar{C}=E\left\{log_{2}\left(1+\gamma_{r}\right)\right\}, \end{array}$$

where $\gamma_{r}$ is the received instantaneous SNR, which is given by $\gamma_{r}=\gamma_{max1}$ in the case where there are no pointing errors. Since the PDFs of (16) and (17) are intractable, we focus on the upper bound of (18) using the well-known Jensen’s inequality, such that

(19) $$\begin{array}{cc} \; & {\bar{C}}_{1}^{UB}=log_{2}\left\{1+E\left(\gamma_{max1}\right)\right\}. \end{array}$$

**Lemma** **1.** *Let the random variables $Z_{2,n}$, $Z_{1,n}$ and $Z_{0}$ denote α-μ distributed variables according to (9), related to the upper bound of the capacity ${\bar{C}}_{1}$ through*

*${\bar{C}}_{1}$ = $log_{2}\left(1+\overset{0}{\gamma }\;E{\left\{\sum_{n=1}^{N}|Z_{2,n}\left|\right|Z_{1,n}\left|+H\right|Z_{0}|\right\}}^{2}\right)$, then ${\bar{C}}_{1}$ can be evaluated as*

(20) $$\begin{array}{cc} {\bar{C}}_{1} & =log_{2}[1+\overset{0}{\gamma }\{N\;\Omega_{2}\;\Omega_{1}+N\left(N-1\right){\left(\frac{\Gamma \left(\mu_{2}+\frac{1}{\alpha_{2}}\right)}{\Gamma \left(\mu_{2}\right)}\right)}^{2}\\ \; & \times {\left(\frac{\Gamma \left(\mu_{1}+\frac{1}{\alpha_{1}}\right)}{\Gamma \left(\mu_{1}\right)}\right)}^{2}\frac{\Omega_{2}\;\Omega_{1}}{\xi_{2}\;\xi_{1}}+H^{2}\Omega_{0}+2\;N\;H\\ \; & \times \frac{\Gamma \left(\mu_{2}+\frac{1}{\alpha_{2}}\right)\Gamma \left(\mu_{1}+\frac{1}{\alpha_{1}}\right)\Gamma \left(\mu_{0}+\frac{1}{\alpha_{0}}\right)}{\Gamma \left(\mu_{2}\right)\Gamma \left(\mu_{1}\right)\Gamma \left(\mu_{0}\right)}\sqrt{\frac{\Omega_{2}\Omega_{1}\Omega_{0}}{\xi_{2}\xi_{1}\xi_{0}}}\}]. \end{array}$$

The derived expression provides a tight upper bound on the ergodic capacity for sufficiently large values of *N*. This is due to the fact that ${\bar{C}}_{1}$ is dominated by the term $N^{2}\overset{0}{\gamma }{\left(\frac{\Gamma \left(\mu_{2}+\frac{1}{\alpha_{2}}\right)}{\Gamma \left(\mu_{2}\right)}\right)}^{2}{\left(\frac{\Gamma \left(\mu_{1}+\frac{1}{\alpha_{1}}\right)}{\Gamma \left(\mu_{1}\right)}\right)}^{2}\frac{\Omega_{2}\;\Omega_{1}}{\xi_{2}\;\xi_{1}}$ providing an SNR gain proportional to $N^{2}$. This fact clearly indicates that the IRS not only yields beamforming gain but also the inherent aperture gain.

**Proof.**  Please see Appendix A. □

### 3.2. Outage Analysis without Pointing Errors

The outage probability is defined as the probability of the received instantaneous SNR falling below the predefined threshold SNR, $\gamma_{th}$. Mathematically, it can be expressed as

(21) $$\begin{array}{cc} P_{out} & :=P_{r}\left[\gamma_{r}<\gamma_{th}\right]\\ \; & =F_{\gamma_{r}}\left(\gamma \right){|}_{\gamma =\gamma_{th}}, \end{array}$$

where $F_{\gamma_{r}}\left(\cdot \right)$ is the CDF of $\gamma_{r}$.

**Lemma** **2.** *Let the random variables considered in Lemma 1 now be related to the outage probability $P_{1}$ through $P_{1}$ = $P_{r}\left[\sum_{n=1}^{N}|Z_{2,n}\left|\right|Z_{1,n}\left|+H\right|Z_{0}|<\sqrt{\frac{\gamma_{th}}{\overset{0}{\gamma }}}\right]$, then $P_{1}$ can be calculated as*

(22) $$\begin{array}{cc} P_{1} & =\frac{1}{2}+\frac{1}{2}\;erf\left(\frac{\sqrt{\frac{\gamma_{th}}{\overset{0}{\gamma }}}-N\mu }{\sqrt{2N\sigma^{2}}}\right)\\ \; & -\frac{exp\left(-\frac{{\left(N\mu -\sqrt{\frac{\gamma_{th}}{\overset{0}{\gamma }}}\right)}^{2}}{2N\sigma^{2}}\right)}{2\sqrt{\pi }\;\Gamma \left(\mu_{0}\right)}\sum_{k=0}^{\infty }\frac{{\left(\sqrt{\frac{2}{N\sigma^{2}}}\left(\sqrt{\frac{\gamma_{th}}{\overset{0}{\gamma }}}-N\mu \right)\right)}^{k}}{k!}\\ \; & \times H_{2,2}^{2,1}\left[\mu_{0}\;{\left(\frac{\sqrt{2N\sigma^{2}}}{H\hat{h_{0}}}\right)}^{\alpha_{0}}|\begin{array}{c} \left(\frac{1}{2}\left(1-k\right),\frac{\alpha_{0}}{2}\right),\left(1,1\right)\\ \left(\mu_{0},1\right),\left(0,1\right) \end{array}\right]. \end{array}$$

It is important to mention here that first 15 terms are sufficient for the convergence of summation inside the proposed expression.

**Proof.**  Please see Appendix B. □

### 3.3. Capacity Analysis with Pointing Errors

Taking advantage of the same concept as followed in Section 3.1, an upper bound of the capacity can be obtained using (17) as

(23) $$\begin{array}{cc} {\bar{C}}_{2}^{UB} & =log_{2}\left(1+E\left\{\gamma_{max2}\right\}\right). \end{array}$$

**Lemma** **3.** *Let $X_{p2,i}$, $X_{p1,i}$ and $X_{p0}$ be the random variables, distributed according to (13), related to the upper bound of capacity ${\bar{C}}_{2}$ through ${\bar{C}}_{2}$ = $log_{2}(1+\overset{0}{\gamma }\;E\{\sum_{i=1}^{N}|X_{p2,i}\left|\right|X_{p1,i}|$$+H|X_{p0}{\left|\right\}}^{2})$, then ${\bar{C}}_{2}$ can be evaluated as*

(24) $$\begin{array}{cc} \; & {\bar{C}}_{2}=\\ \; & log_{2}[1+\overset{0}{\gamma }\{N\;\xi_{2}\xi_{1}\left(\frac{\phi_{2}}{2+\phi_{2}}\right)\left(\frac{\phi_{1}}{2+\phi_{1}}\right){\left(\frac{S_{2}\;\hat{h_{2}}}{\mu_{2}^{\frac{1}{\alpha_{2}}}}\right)}^{2}{\left(\frac{S_{1}\;\hat{h_{1}}}{\mu_{1}^{\frac{1}{\alpha_{1}}}}\right)}^{2}\\ \; & +N\left(N-1\right){\left(\frac{\phi_{2}}{1+\phi_{2}}\times \frac{S_{2}\;\hat{h_{2}}}{\mu_{2}^{\frac{1}{\alpha_{2}}}}\times \frac{\Gamma \left(\mu_{2}+\frac{1}{\alpha_{2}}\right)}{\Gamma \left(\mu_{2}\right)}\right)}^{2}\\ \; & \times {\left(\frac{\phi_{1}}{1+\phi_{1}}\times \frac{S_{1}\;\hat{h_{1}}}{\mu_{1}^{\frac{1}{\alpha_{1}}}}\times \frac{\Gamma \left(\mu_{1}+\frac{1}{\alpha_{1}}\right)}{\Gamma \left(\mu_{1}\right)}\right)}^{2}+H^{2}\;\xi_{0}\left(\frac{\phi_{0}}{2+\phi_{0}}\right){\left(\frac{S_{0}\;\hat{h_{0}}}{\mu_{0}^{\frac{1}{\alpha_{0}}}}\right)}^{2}\\ \; & +2\;N\;H\;\left(\frac{\phi_{2}}{1+\phi_{2}}\right)\left(\frac{\phi_{1}}{1+\phi_{1}}\right)\left(\frac{\phi_{0}}{1+\phi_{0}}\right)\left(\frac{S_{2}\;\hat{h_{2}}}{\mu_{2}^{\frac{1}{\alpha_{2}}}}\right)\left(\frac{S_{1}\;\hat{h_{1}}}{\mu_{1}^{\frac{1}{\alpha_{1}}}}\right)\\ \; & \times \left(\frac{S_{0}\;\hat{h_{0}}}{\mu_{0}^{\frac{1}{\alpha_{0}}}}\right)\frac{\Gamma \left(\mu_{2}+\frac{1}{\alpha_{2}}\right)\Gamma \left(\mu_{1}+\frac{1}{\alpha_{1}}\right)\Gamma \left(\mu_{0}+\frac{1}{\alpha_{0}}\right)}{\Gamma \left(\mu_{2}\right)\Gamma \left(\mu_{1}\right)\Gamma \left(\mu_{0}\right)}\}]. \end{array}$$

**Proof.**  Please see Appendix C. □

### 3.4. Outage Analysis with Pointing Errors

The probability of an outage can also be written by using the same concept as explained in Section 3.2.

**Lemma** **4.** *Let the random variables considered in Lemma 3 now be related to the outage probability $P_{2}$ through $P_{2}$ = $P_{r}\left[\sum_{i=1}^{N}|X_{2,i}\left|\right|X_{1,i}\left|+H\right|X_{0}|<\sqrt{\frac{\gamma_{th}}{\overset{0}{\gamma }}}\right]$, then $P_{2}$ can be calculated as*

(25) $$\begin{array}{cc} P_{2} & =\frac{1}{2}+\frac{1}{2}\;erf\left(\frac{\sqrt{\frac{\gamma_{th}}{\overset{0}{\gamma }}}-N\mu }{\sqrt{2N\sigma^{2}}}\right)-\frac{exp\left(-\frac{{\left(N\mu -\sqrt{\frac{\gamma_{th}}{\overset{0}{\gamma }}}\right)}^{2}}{2N\sigma^{2}}\right)}{\sqrt{2\pi N\sigma^{2}}}\\ \; & \times \frac{\phi_{0}{\left(2N\sigma^{2}\right)}^{\frac{\phi_{0}+1}{2}}}{2\alpha_{0}{\left(S_{0}\;\hat{h_{0}}\;H\right)}^{\phi_{0}}}\sum_{k=0}^{\mu_{0}-1}\frac{{\left(\mu_{0}\right)}^{\frac{\phi_{0}}{\alpha_{0}}}}{k!}\\ \; & \times \sum_{n=0}^{\infty }\frac{{\left(\sqrt{\frac{2}{N\sigma^{2}}}\left(\sqrt{\frac{\gamma_{th}}{\overset{0}{\gamma }}}-N\mu \right)\right)}^{n}}{n!}\\ \; & \times H_{2,2}^{2,1}\left[\mu_{0}\;{\left(\frac{\sqrt{2N\sigma^{2}}}{S_{0}\;\hat{h_{0}}\;H}\right)}^{\alpha_{0}}|\begin{array}{c} \left(\frac{1}{2}\left(1-\phi_{0}-n\right),\frac{\alpha_{0}}{2}\right),\left(1,1\right)\\ \left(k-\frac{\phi_{0}}{\alpha_{0}},1\right),\left(0,1\right) \end{array}\right]. \end{array}$$

It is important to mention here that only the first 15 terms are sufficient for the convergence of summation inside the proposed expression.

**Proof.**  Please see Appendix D. □

## 4. Results and Discussion

The derived analytical expressions for the ergodic capacity and outage probability in Section 3 are validated in this section through numerical Monte Carlo simulations. The α-μ fading channel parameters for the IRS links are $\alpha_{1}=\alpha_{2}=2$, $\mu_{1}=\mu_{2}=5.76$ and $\Omega_{1}=\Omega_{2}=1$, which according to the experiments carried out in [4] corresponds to a LoS scenario. Moreover, for the direct link, we use $\alpha_{0}=0.5$, $\mu_{0}=0.5$ and $\Omega_{0}=1$. The distance between the Tx–Rx link is computed as $d_{0}$ = $\sqrt{d_{1}^{2}+d_{2}^{2}-2d_{1}d_{2}\cos\left(\theta \right)}$ with $\theta =60^{0}$, where $d_{1}$ and $d_{2}$ are the distances of the Tx–IRS and IRS–Rx links, respectively. Here, we assume that $d_{1}=d_{2}=d$ and $G_{t}=G_{r}=25$ dB. The bandwidth, *B*, of THz signal and carrier frequency, *f*, are set to 10 GHz and 275 GHz, respectively. The SNR is given by $\frac{P_{t}}{BN_{0}}$, where $N_{0}=-173$ dBm/Hz. The results plotted in this section are averaged over 10,000 independent channel realizations.

### 4.1. Without Pointing Error

**Figure 2:**
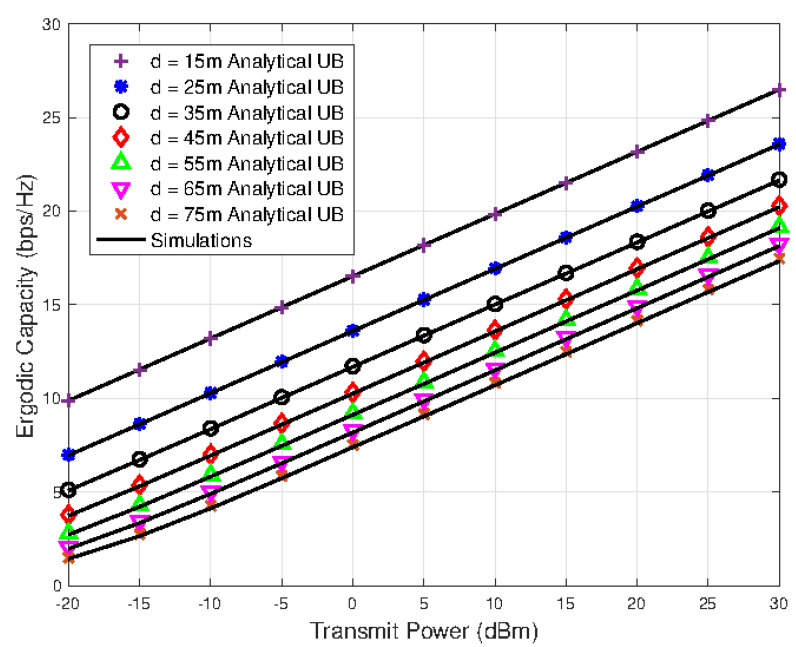
Ergodic capacity versus transmit power results for various values of the link distance.

**Figure 3:**
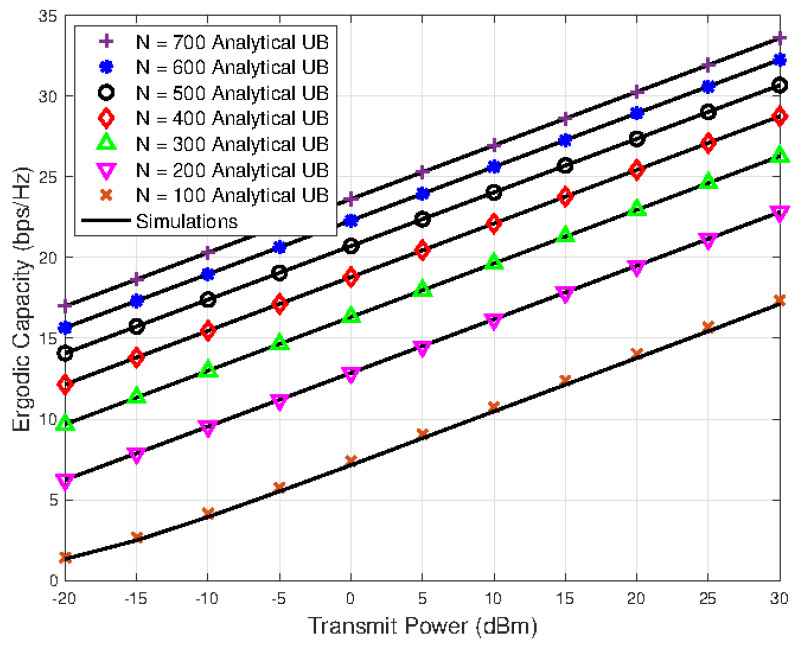
Ergodic capacity versus transmit power results with different numbers of reflecting elements.

Figure 2 shows the ergodic capacity results against different transmit power, *P*, and distance, *d*, values for the fixed number of IRS elements i.e., N=128. It can be seen that the ergodic capacity results are superior for the scenarios having smaller link distances. In Figure 3, the ergodic capacity of the system is evaluated for various levels of the transmit power and different values of IRS reflecting elements *N*. As the value of *N* increases, the ergodic capacity also increases; however, it can be seen that the performance gap slowly shrinks for higher values of *N*. The tightness of the derived upper bounded ergodic capacity analytical expression is also evident from the results plotted in Figure 2 and Figure 3.

**Figure 4:**
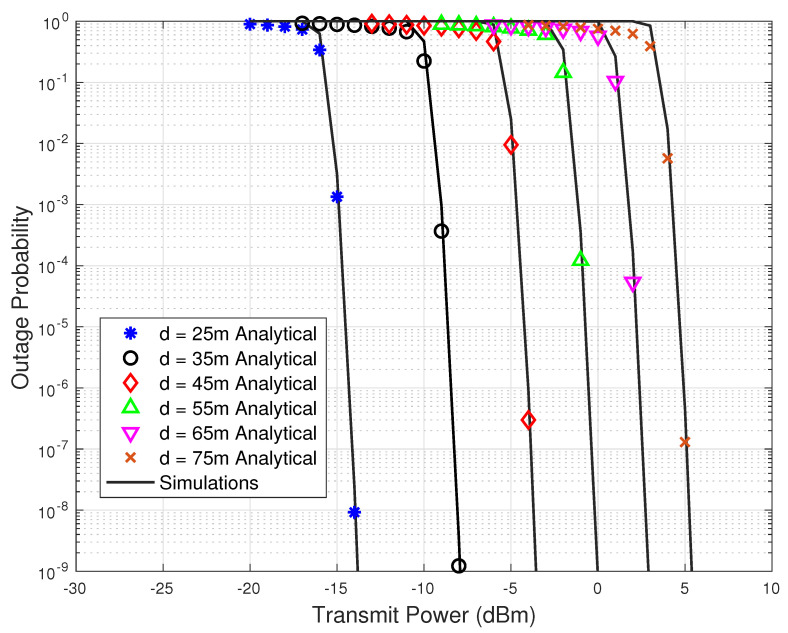
Outage probability versus transmit power results for various values of the link distance.

**Figure 5:**
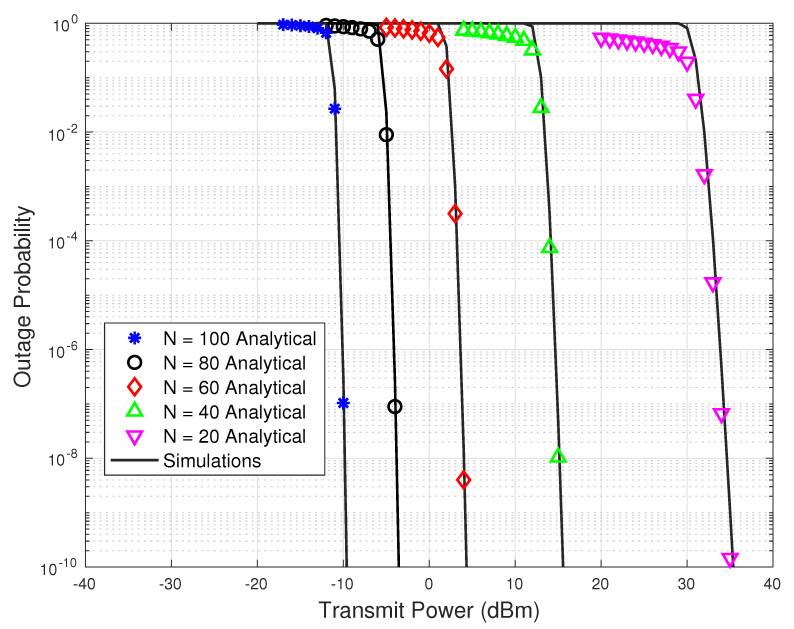
Outage probability versus transmit power results with different numbers of reflecting elements.

Figure 4 and Figure 5 show the outage probability versus transmit power results for different values of link distances, *d*, and number of IRS reflecting elements, *N*, respectively. In Figure 4, the total number of reflecting elements in the IRS are set to N=70. From Figure 4, it is evident that the outage performance improves significantly as the link distance decreases. From the network design perspective, to reduce the total transmission power of the network, the link distances should be kept small in THz systems. Similarly, from Figure 5, a gain of about 25 dBm in the transmit power is observed when the number of reflecting elements is increased from N=40 to N=100.

### 4.2. With Pointing Error

**Figure 6:**
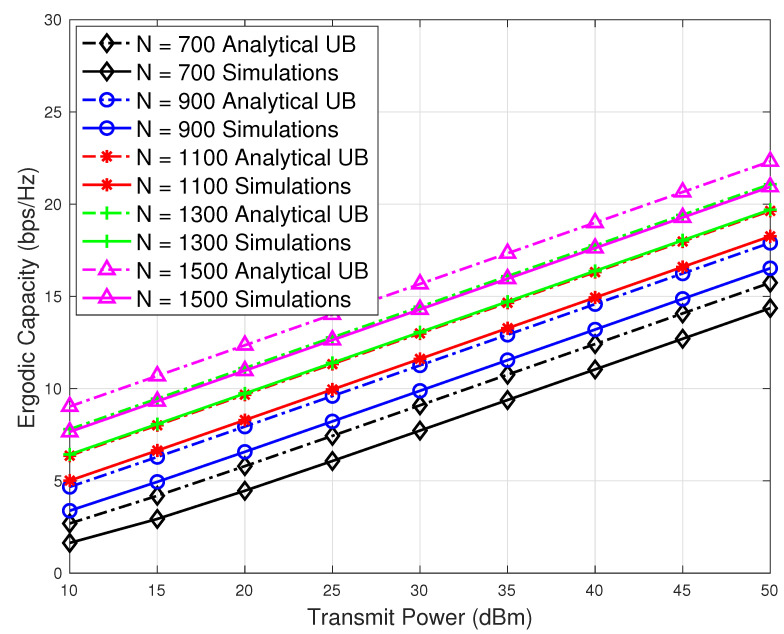
Ergodic capacity with pointing error versus transmit power results with different numbers of reflecting elements.

As mentioned earlier for the system with pointing error, the number of reflecting elements should be considerably large. Therefore, we plotted the results for the large values of reflecting elements *N*. In Figure 6, we plot the ergodic capacity results against various transmission power and *N* values. Here, we also include the effects of pointing errors on the ergodic capacity. It can be seen that ergodic capacity decreases significantly in the presence of pointing error compared to the case when pointing errors are not present. Therefore, the increase in the number of reflecting elements does not show remarkable performance improvement, as shown in Figure 3 (without pointing errors). From Figure 6, it is evident that our analytical upper bound expressions on ergodic capacity become tight as the value of *N* increases.

**Figure 7:**
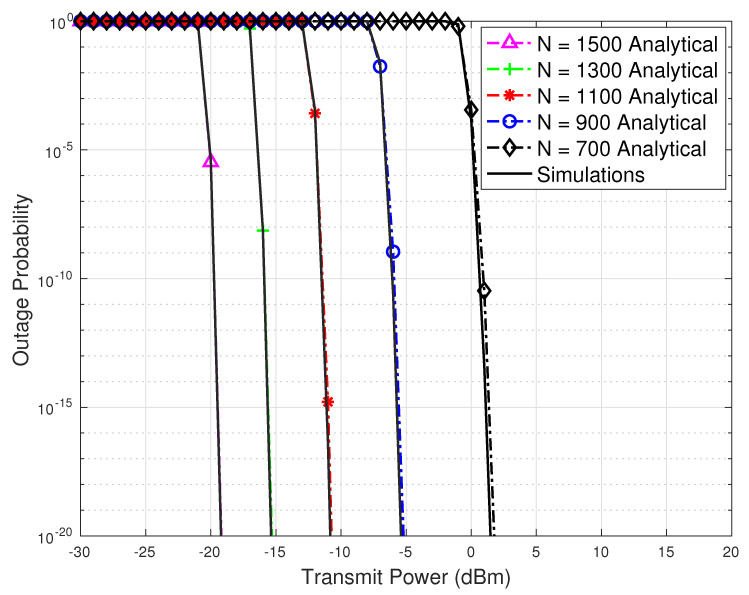
Outage probability with pointing error versus transmit power results with different numbers of reflecting elements.

For the same values of *N* as in Figure 6, in Figure 7, we plot the outage probability against different transmission power values in the presence of pointing error. It has been observed that a large number of reflecting elements are required to achieve an outage probability that is comparable with the outage probability without pointing error. While comparing Figure 5 and Figure 7, it can be seen that to achieve a comparable performance, the number of reflecting elements in the case of pointing error should be almost 10 times more than the case without pointing errors. Moreover, the analytical results plotted in Figure 7 match well with the simulation-based numerical results.

## 5. Conclusions

The performance of the IRS-assisted THz wireless communication system has been analyzed in terms of ergodic capacity and outage probability. By using the statistical distribution of the α-μ fading channel, the upper bound on the ergodic capacity and the approximation of the outage probability are derived. These analytical closed-form expressions are validated by a rigorous set of numerical simulations. It has been observed that to improve the coverage in THz systems, an IRS with massive reflecting elements should be deployed. Moreover, it has been observed that increasing the number of reflecting elements in the IRS results not only in coverage enhancement but also improves the power consumption. It is important to mention here that pointing errors have a detrimental effect on the performance of the THz communication system. For example, it has been noticed that to achieve a comparable performance, the number of reflecting elements in the presence of pointing errors should be almost 10 times more than the case without pointing errors.

## Data Availability

Not applicable.

## Appendix A. Proof of Lemma 1

Let ${\bar{C}}_{1}$ be re-written as

(A1) $$\begin{array}{cc} {\bar{C}}_{1} & =log_{2}[1+\overset{0}{\gamma }\{\underset{S_{1}}{\underset{︸}{E{\left(\sum_{n=1}^{N}\left\{|Z_{2,n}\left|\right|Z_{1,n}|\right\}\right)}^{2}}}\\ \; & +H^{2}\underset{S_{2}}{\underset{︸}{E\left\{|Z_{0}{|}^{2}\right\}}}\\ \; & +2H\underset{S_{3}}{\underset{︸}{E\left\{|Z_{0}|\right\}}}\sum_{n=1}^{N}\underset{S_{4}}{\underset{︸}{E\left\{|Z_{2,n}|\right\}|}}\underset{S_{5}}{\underset{︸}{E\left\{Z_{1,n}|\right\}}}\}]. \end{array}$$

Here we have exploited the statistical independence of $|Z_{0}|$, $|Z_{1,n}|$ and $|Z_{2,n}|$ in writing the second and third part of (A1). Now the first term $S_{1}$ can be re-written as

(A2) $$\begin{array}{cc} S_{1} & =\underset{S_{11}}{\underset{︸}{E\left\{\sum_{n=1}^{N}|Z_{2,n}{|}^{2}{\left|Z_{1,n}\right|}^{2}\right\}}}\\ \; & +\underset{S_{12}}{\underset{︸}{E\left\{\sum_{n=1}^{N}\sum_{i=1;i\neq n}^{N}|Z_{2,n}\left|\right|Z_{1,n}\left|\right|Z_{2,i}\left|\right|Z_{1,i}|\right\}}}. \end{array}$$

Now $S_{11}$ evaluates to

(A3) $$\begin{array}{cc} S_{11} & =\sum_{n=1}^{N}E\left\{|Z_{2,n}{|}^{2}\right\}\;E\left\{|Z_{1,n}{|}^{2}\right\}\\ \; & =N\;\Omega_{2}\;\Omega_{1}. \end{array}$$

Since the vectors $Z_{2}$ and $Z_{1}$ are statistically independent as observed in $S_{12}$, therefore $S_{12}$ results in

(A4) $$\begin{array}{cc} S_{12} & =N\left(N-1\right)\times {\left(\frac{\Gamma \left(\mu_{2}+\frac{1}{\alpha_{2}}\right)}{\Gamma \left(\mu_{2}\right)}\right)}^{2}\\ \; & \times {\left(\frac{\Gamma \left(\mu_{1}+\frac{1}{\alpha_{1}}\right)}{\Gamma \left(\mu_{1}\right)}\right)}^{2}\times \frac{\Omega_{2}\;\Omega_{1}}{\xi_{2}\;\xi_{1}}. \end{array}$$

Similarly, we obtain $S_{2}$ = $\Omega_{0}$, $S_{3}$ = $\frac{\Gamma \left(\mu_{0}+\frac{1}{\alpha_{0}}\right)}{\Gamma \left(\mu_{0}\right)}\sqrt{\frac{\Omega_{0}}{\xi_{0}}}$, $S_{4}$ = $\frac{\Gamma \left(\mu_{2}+\frac{1}{\alpha_{2}}\right)}{\Gamma \left(\mu_{2}\right)}$$\sqrt{\frac{\Omega_{2}}{\xi_{2}}}$ and $S_{5}$ = $\frac{\Gamma \left(\mu_{1}+\frac{1}{\alpha_{1}}\right)}{\Gamma \left(\mu_{1}\right)}\sqrt{\frac{\Omega_{1}}{\xi_{1}}}$, respectively. Substituting the values of $S_{1}$-$S_{5}$ in (A1), we obtain (20), and hence the proof is completed. □

## Appendix B. Proof of Lemma 2

Let $P_{1}$ be expressed as

(A5) $$\begin{array}{c} P_{1}=P_{r}\left[z<\sqrt{\frac{\gamma_{th}}{\overset{0}{\gamma }}}\right], \end{array}$$

where $z\overset{△}{=}u+v$ in which *u* = $\sum_{n=1}^{N}|Z_{2,n}\left|\right|Z_{1,n}|$ and *v* = $H|Z_{0}|$. According to central limit theorem, *u* can be well approximated by normal distribution with mean μ and variance $\sigma^{2}$ provided *N* is sufficiently large, i.e., $u∼N\left(N\mu ,N\sigma^{2}\right)$.

(A6) $$\begin{array}{cc} \mu & =E\left\{|Z_{2,n}\left|\right|Z_{1,n}|\right\}\\ \; & =\frac{\Gamma \left(\mu_{2}+\frac{1}{\alpha_{2}}\right)\Gamma \left(\mu_{1}+\frac{1}{\alpha_{1}}\right)}{\Gamma \left(\mu_{2}\right)\Gamma \left(\mu_{1}\right)}\sqrt{\frac{\Omega_{2}\Omega_{1}}{\xi_{2}\xi_{1}}}. \end{array}$$

(A7) $$\begin{array}{cc} \sigma^{2} & =Var\left\{|Z_{2,n}\left|\right|Z_{1,n}|\right\}\\ \; & =\Omega_{2}\Omega_{1}\left\{1-\frac{1}{\xi_{2}\xi_{1}}{\left(\frac{\Gamma \left(\mu_{2}+\frac{1}{\alpha_{2}}\right)\Gamma \left(\mu_{1}+\frac{1}{\alpha_{1}}\right)}{\Gamma \left(\mu_{2}\right)\Gamma \left(\mu_{1}\right)}\right)}^{2}\right\}. \end{array}$$

The PDF of *u* would become

(A8) $$\begin{array}{c} f_{u}\left(x\right)\underset{N\to \infty }{\approx }\frac{1}{\sqrt{2\pi \;N\sigma^{2}}}\;exp\left[-\frac{{\left(x-N\mu \right)}^{2}}{2N\sigma^{2}}\right]. \end{array}$$

The CDF of *v* can be written as

(A9) $$\begin{array}{c} F_{v}\left(x\right)=1-\frac{1}{\Gamma \left(\mu_{0}\right)}\;\Gamma \left(\mu_{0},\frac{\mu_{0}}{{\left(H\hat{h_{0}}\right)}^{\alpha_{0}}}\;x^{\alpha_{0}}\right). \end{array}$$

Benefiting from the basic laws of statistics, the CDF of the sum of two independent random variables can be written as

(A10) $$\begin{array}{c} F_{z}\left(z\right)\underset{N\to \infty }{\approx }\int_{-\infty }^{\infty }f_{u}\left(z-x\right)\;F_{v}\left(x\right)\;dx. \end{array}$$

Substituting (A8) and (A9) in (A10), along with some simplifications, we obtain

(A11) $$\begin{array}{cc} \; & F_{z}\left(z\right)\underset{N\to \infty }{\approx }\underset{I_{1}}{\underset{︸}{A\int_{0}^{\infty }exp\left(-\frac{x^{2}}{2N\sigma^{2}}+\frac{\left(z-N\mu \right)}{N\sigma^{2}}x\right)\;dx}}\\ \; & -\underset{I_{2}}{\underset{︸}{B\;\int_{0}^{\infty }\;exp\left(-\frac{x^{2}}{2N\sigma^{2}}+\frac{\left(z-N\mu \right)}{N\sigma^{2}}x\right)\;\Gamma \;\left(\mu_{0},\frac{\mu_{0}}{{\left(H\hat{h_{0}}\right)}^{\alpha_{0}}}\;x^{\alpha_{0}}\;\right)\;dx}}, \end{array}$$

where *A* = $\frac{exp\left(-\frac{{\left(N\mu -z\right)}^{2}}{2N\sigma^{2}}\right)}{\sqrt{2\pi \;N\sigma^{2}}}$ and *B* = $\frac{A}{\Gamma \left(\mu_{0}\right)}$. The integral $I_{1}$ can be evaluated using [15] Equation (3.322.2) as

(A12) $$\begin{array}{c} I_{1}=\frac{1}{2}+\frac{1}{2}\;erf\left(\frac{z-N\mu }{\sqrt{2N\sigma^{2}}}\right), \end{array}$$

where $erf\left(\cdot \right)$ is the error function [15], Equation (8.250.1). The integral $I_{2}$ can be re-written by expressing $exp\left(\frac{\left(z-N\mu \right)}{N\sigma^{2}}x\right)$ in series as

(A13) $$\begin{array}{cc} I_{2} & =\frac{exp\left(-\frac{{\left(N\mu -z\right)}^{2}}{2N\sigma^{2}}\right)}{\sqrt{2\pi \;N\sigma^{2}}\;\Gamma \left(\mu_{0}\right)}\sum_{k=0}^{\infty }\frac{{\left(\frac{\left(z-N\mu \right)}{N\sigma^{2}}\right)}^{k}}{k!}\\ \; & \times \underset{I_{22}}{\underset{︸}{\int_{0}^{\infty }x^{k}\;exp\left(-\frac{x^{2}}{2N\sigma^{2}}\right)\Gamma \left(\mu_{0},\frac{\mu_{0}}{{\left(H\hat{h_{0}}\right)}^{\alpha_{0}}}\;x^{\alpha_{0}}\right)\;dx}}. \end{array}$$

To evaluate $I_{22}$, we express $exp\left(-\frac{x^{2}}{2N\sigma^{2}}\right)$ and $\Gamma \left(\cdot ,\cdot \right)$ in terms of Fox’s H-function using [17] Equations (1.7.2) and (1.2.3) and [18], respectively. Now $I_{22}$ would be

(A14) $$\begin{array}{cc} I_{22} & =\frac{1}{2}\int_{0}^{\infty }x^{k}H_{1,2}^{2,0}\left[\frac{\mu_{0}}{{\left(H\hat{h_{0}}\right)}^{\alpha_{0}}}\;x^{\alpha_{0}}|\begin{array}{c} \left(1,1\right)\\ \left(\mu_{0},1\right),\left(0,1\right) \end{array}\right]\\ \; & \times H_{0,1}^{1,0}\left[\sqrt{\frac{1}{2N\sigma^{2}}}x|\begin{array}{c} ¯\\ \left(0,\frac{1}{2}\right) \end{array}\right]\;dx. \end{array}$$

Making substitution of $\sqrt{\frac{1}{2N\sigma^{2}}}x$ = *u*, $I_{22}$ would become

(A15) $$\begin{array}{cc} I_{22} & =\frac{1}{2}{\left(2N\sigma^{2}\right)}^{\frac{k+1}{2}}\int_{0}^{\infty }u^{k}\\ \; & \times H_{1,2}^{2,0}\left[\mu_{0}\;{\left(\frac{\sqrt{2N\sigma^{2}}}{H\hat{h_{0}}}\right)}^{\alpha_{0}}u^{\alpha_{0}}|\begin{array}{c} \left(1,1\right)\\ \left(\mu_{0},1\right),\left(0,1\right) \end{array}\right]\\ \; & \times H_{0,1}^{1,0}\left[u|\begin{array}{c} ¯\\ \left(0,\frac{1}{2}\right) \end{array}\right]\;du. \end{array}$$

$I_{22}$ can be solved using [17] Equation (2.6.8) as

(A16) $$\begin{array}{cc} I_{22} & =\frac{1}{2}{\left(2N\sigma^{2}\right)}^{\frac{k+1}{2}}\\ \; & \times H_{2,2}^{2,1}\left[\mu_{0}\;{\left(\frac{\sqrt{2N\sigma^{2}}}{H\hat{h_{0}}}\right)}^{\alpha_{0}}|\;\begin{array}{c} \left(\frac{1}{2}\left(1-k\right),\frac{\alpha_{0}}{2}\right),\left(1,1\right)\\ \left(\mu_{0},1\right),\left(0,1\right) \end{array}\;\right]. \end{array}$$

Substituting $I_{22}$ in (A13), we obtain $I_{2}$ as

(A17) $$\begin{array}{cc} I_{2} & =\frac{exp\left(-\frac{{\left(N\mu -z\right)}^{2}}{2N\sigma^{2}}\right)}{2\sqrt{\pi }\;\Gamma \left(\mu_{0}\right)}\sum_{k=0}^{\infty }\frac{{\left(\sqrt{\frac{2}{N\sigma^{2}}}\left(z-N\mu \right)\right)}^{k}}{k!}\\ \; & \times H_{2,2}^{2,1}\left[\mu_{0}\;{\left(\frac{\sqrt{2N\sigma^{2}}}{H\hat{h_{0}}}\right)}^{\alpha_{0}}|\;\begin{array}{c} \left(\frac{1}{2}\left(1-k\right),\frac{\alpha_{0}}{2}\right),\left(1,1\right)\\ \left(\mu_{0},1\right),\left(0,1\right) \end{array}\;\right]. \end{array}$$

By putting $I_{1}$ and $I_{2}$ in (A11), we obtain the desired result and complete the proof. □

## Appendix C. Proof of Lemma 3

Let ${\bar{C}}_{2}$ be re-written as

(A18) $$\begin{array}{cc} {\bar{C}}_{2} & =log_{2}[1+\overset{0}{\gamma }\{\underset{E_{1}}{\underset{︸}{E{\left(\sum_{i=1}^{N}\left\{|X_{p2,i}\left|\right|X_{p1,i}|\right\}\right)}^{2}}}\\ \; & +H^{2}\underset{E_{2}}{\underset{︸}{E\left\{|X_{p0}{|}^{2}\right\}}}\\ \; & +2H\underset{E_{3}}{\underset{︸}{E\left\{|X_{p0}|\right\}}}\sum_{i=1}^{N}\underset{E_{4}}{\underset{︸}{E\left\{|X_{p2,i}|\right\}}}\underset{E_{5}}{\underset{︸}{E\left\{X_{p1,i}|\right\}}}\}]. \end{array}$$

It may be noted that $|X_{p0}|$, $|X_{p1,i}|$ and $|X_{p2,i}|$ are statistically independent and this property has been used in the second and third part of (A18). To evaluate $E_{1}$, we re-write it as

(A19) $$\begin{array}{cc} E_{1} & =\underset{E_{11}}{\underset{︸}{E\left\{\sum_{i=1}^{N}|X_{p2,i}{|}^{2}{\left|X_{p1,i}\right|}^{2}\right\}}}\\ \; & +\underset{E_{12}}{\underset{︸}{E\left\{\sum_{i=1}^{N}|X_{p2,i}\left|\right|X_{p1,i}|\sum_{j=1;j\neq i}^{N}|X_{p2,j}\left|\right|X_{p1,j}|\right\}}}. \end{array}$$

Now $E_{11}$ evaluates to

(A20) $$\begin{array}{cc} E_{11} & =\sum_{i=1}^{N}E\left\{|X_{p2,i}{|}^{2}\right\}\;E\left\{|X_{p1,i}{|}^{2}\right\}\\ \; & =N\;\xi_{2}\xi_{1}\left(\frac{\phi_{2}}{2+\phi_{2}}\right)\left(\frac{\phi_{1}}{2+\phi_{1}}\right){\left(\frac{S_{2}\;\hat{h_{2}}}{\mu_{2}^{\frac{1}{\alpha_{2}}}}\right)}^{2}{\left(\frac{S_{1}\;\hat{h_{1}}}{\mu_{1}^{\frac{1}{\alpha_{1}}}}\right)}^{2}. \end{array}$$

$E_{12}$ can be evaluated by considering the statistical independence of the vectors $X_{2}$ and $X_{1}$, and therefore $E_{12}$ would become

(A21) $$\begin{array}{cc} E_{12} & =N\left(N-1\right)\times [\left(\frac{\phi_{2}}{1+\phi_{2}}\right)\left(\frac{S_{2}\;\hat{h_{2}}}{\mu_{2}^{\frac{1}{\alpha_{2}}}}\right)\frac{\Gamma \left(\mu_{2}+\frac{1}{\alpha_{2}}\right)}{\Gamma \left(\mu_{2}\right)}\\ \; & \times \left(\frac{\phi_{1}}{1+\phi_{1}}\right)\left(\frac{S_{1}\;\hat{h_{1}}}{\mu_{1}^{\frac{1}{\alpha_{1}}}}\right)\frac{\Gamma \left(\mu_{1}+\frac{1}{\alpha_{1}}\right)}{\Gamma \left(\mu_{1}\right)}{]}^{2}. \end{array}$$

Similarly, we obtain $E_{2}$ = $\xi_{0}\left(\frac{\phi_{0}}{2+\phi_{0}}\right){\left(\frac{S_{0}\;\hat{h_{0}}}{\mu_{0}^{\frac{1}{\alpha_{0}}}}\right)}^{2}$, $E_{3}$ = $\left(\frac{\phi_{0}}{1+\phi_{0}}\right)$$\left(\frac{S_{0}\;\hat{h_{0}}}{\mu_{0}^{\frac{1}{\alpha_{0}}}}\right)\frac{\Gamma \left(\mu_{0}+\frac{1}{\alpha_{0}}\right)}{\Gamma \left(\mu_{0}\right)}$,

$E_{4}$ = $\left(\frac{\phi_{2}}{1+\phi_{2}}\right)\left(\frac{S_{2}\;\hat{h_{2}}}{\mu_{2}^{\frac{1}{\alpha_{2}}}}\right)\frac{\Gamma \left(\mu_{2}+\frac{1}{\alpha_{2}}\right)}{\Gamma \left(\mu_{2}\right)}$ and $E_{5}$ = $\left(\frac{\phi_{1}}{1+\phi_{1}}\right)\left(\frac{S_{1}\;\hat{h_{1}}}{\mu_{1}^{\frac{1}{\alpha_{1}}}}\right)\frac{\Gamma \left(\mu_{1}+\frac{1}{\alpha_{1}}\right)}{\Gamma \left(\mu_{1}\right)}$, respectively.

Substituting the values of $E_{1}$–$E_{5}$ in (A18), we obtain (24) and hence the proof is completed. □

## Appendix D. Proof of Lemma 4

Let $P_{2}$ be written as

(A22) $$\begin{array}{c} P_{2}=P_{r}\left[x<\sqrt{\frac{\gamma_{th}}{\overset{0}{\gamma }}}\right], \end{array}$$

where $x\overset{△}{=}u+v$ in which *u* = $\sum_{i=1}^{N}|X_{p2,i}\left|\right|X_{p1,i}|$ and *v* = $H|X_{0}|$. Once again, we take advantage of the central limit theorem and approximate the random variable $u∼N\left(N\mu ,N\sigma^{2}\right)$ with mean μ and variance $\sigma^{2}$ as normally distributed subject to sufficiently large *N*.

(A23) $$\begin{array}{cc} \mu & =E\left\{|X_{p2,i}\left|\right|X_{p1,i}|\right\}\\ \; & =\left(\frac{\phi_{2}}{1+\phi_{2}}\right)\left(\frac{S_{2}\;\hat{h_{2}}}{\mu_{2}^{\frac{1}{\alpha_{2}}}}\right)\left(\frac{\Gamma \left(\mu_{2}+\frac{1}{\alpha_{2}}\right)}{\Gamma \left(\mu_{2}\right)}\right)\\ \; & \times \left(\frac{\phi_{1}}{1+\phi_{1}}\right)\left(\frac{S_{1}\;\hat{h_{1}}}{\mu_{1}^{\frac{1}{\alpha_{1}}}}\right)\left(\frac{\Gamma \left(\mu_{1}+\frac{1}{\alpha_{1}}\right)}{\Gamma \left(\mu_{1}\right)}\right). \end{array}$$

(A24) $$\begin{array}{cc} \sigma^{2} & =Var\left\{|X_{p2,i}\left|\right|X_{p1,i}|\right\}\\ \; & =\xi_{2}\xi_{1}\left(\frac{\phi_{2}}{2+\phi_{2}}\right)\left(\frac{\phi_{1}}{2+\phi_{1}}\right){\left(\frac{S_{2}\;\hat{h_{2}}}{\mu_{2}^{\frac{1}{\alpha_{2}}}}\right)}^{2}{\left(\frac{S_{1}\;\hat{h_{1}}}{\mu_{1}^{\frac{1}{\alpha_{1}}}}\right)}^{2}\\ \; & -{\left(\frac{\phi_{2}}{1+\phi_{2}}\times \frac{S_{2}\;\hat{h_{2}}}{\mu_{2}^{\frac{1}{\alpha_{2}}}}\times \frac{\Gamma \left(\mu_{2}+\frac{1}{\alpha_{2}}\right)}{\Gamma \left(\mu_{2}\right)}\right)}^{2}\\ \; & \times {\left(\frac{\phi_{1}}{1+\phi_{1}}\times \frac{S_{1}\;\hat{h_{1}}}{\mu_{1}^{\frac{1}{\alpha_{1}}}}\times \frac{\Gamma \left(\mu_{1}+\frac{1}{\alpha_{1}}\right)}{\Gamma \left(\mu_{1}\right)}\right)}^{2}. \end{array}$$

The CDF of *v* can be expressed as

(A25) $$\begin{array}{cc} F_{v}\left(t\right) & =1-\frac{\phi_{0}\;t^{\phi_{0}}}{\alpha_{0}{\left(S_{0}\;\hat{h_{0}}\;H\right)}^{\phi_{0}}}\\ \; & \times \sum_{k=0}^{\mu_{0}-1}\frac{{\left(\mu_{0}\right)}^{\frac{\phi_{0}}{\alpha_{0}}}}{k!}\Gamma \left(k-\frac{\phi_{0}}{\alpha_{0}},\frac{\mu_{0}\;t^{\alpha_{0}}}{{\left(S_{0}\;\hat{h_{0}\;H}\right)}^{\alpha_{0}}}\right). \end{array}$$

It is pertinent to mention here that the expression (A8) can be used in the proposed lemma without any modification.

The CDF of the sum of two independent random variables can be re-written as

(A26) $$\begin{array}{c} F_{X}\left(x\right)\underset{N\to \infty }{\approx }\int_{-\infty }^{\infty }f_{u}\left(x-t\right)\;F_{v}\left(t\right)\;dt \end{array}$$

We substitute (A8) and (A25) in (A26) and after some simplifications, we obtain

(A27) $$\begin{array}{cc} \; & F_{X}\left(x\right)\underset{N\to \infty }{\approx }\underset{I_{3}}{\underset{︸}{A\int_{0}^{\infty }exp\left(-\frac{t^{2}}{2N\sigma^{2}}-\frac{\left(N\mu -x\right)}{N\sigma^{2}}t\right)\;dt}}\\ \; & -\frac{A\;\times \phi_{0}}{\alpha_{0}{\left(S_{0}\;\hat{h_{0}}\;H\right)}^{\phi_{0}}}\sum_{k=0}^{\mu_{0}-1}\frac{{\left(\mu_{0}\right)}^{\frac{\phi_{0}}{\alpha_{0}}}}{k!}\\ \; & \times \;\underset{I_{4}}{\underset{︸}{\int_{0}^{\infty }\;t^{\phi_{0}}exp\left(\frac{\left(x-N\mu \right)}{N\sigma^{2}}t-\frac{t^{2}}{2N\sigma^{2}}\right)\;\Gamma \;\left(k-\frac{\phi_{0}}{\alpha_{0}},\frac{\mu_{0}\;t^{\alpha_{0}}}{{\left(S_{0}\;\hat{h_{0}\;H}\right)}^{\alpha_{0}}}\right)\;dt}}, \end{array}$$

where *A* = $\frac{exp\left(-\frac{{\left(N\mu -x\right)}^{2}}{2N\sigma^{2}}\right)}{\sqrt{2\pi \;N\sigma^{2}}}$. The integral $I_{3}$ can be evaluated using [15] Equation (3.322.2) as

(A28) $$\begin{array}{c} I_{3}=\frac{1}{2}+\frac{1}{2}\;erf\left(\frac{x-N\mu }{\sqrt{2N\sigma^{2}}}\right), \end{array}$$

where $erf\left(\cdot \right)$ is the error function [15], Equation (8.250.1). The integral $I_{4}$ can be re-written by expressing $exp\left(\frac{\left(x-N\mu \right)}{N\sigma^{2}}t\right)$ in series as

(A29) $$\begin{array}{cc} I_{4} & =\sum_{n=0}^{\infty }\frac{{\left(\frac{\left(x-N\mu \right)}{N\sigma^{2}}\right)}^{n}}{n!}\int_{0}^{\infty }t^{\phi_{0}+n}\;exp\left(-\frac{t^{2}}{2N\sigma^{2}}\right)\\ \; & \times \Gamma \;\left(k-\frac{\phi_{0}}{\alpha_{0}},\frac{\mu_{0}\;t^{\alpha_{0}}}{{\left(S_{0}\;\hat{h_{0}\;H}\right)}^{\alpha_{0}}}\right)\;dt. \end{array}$$

To evaluate $I_{4}$, we express $exp\left(-\frac{x^{2}}{2N\sigma^{2}}\right)$ and $\Gamma \left(\cdot ,\cdot \right)$ in terms of Fox’s H-function using [17], Equations (1.7.2) and (1.2.3), and [18], respectively. Now $I_{4}$ would be

(A30) $$\begin{array}{cc} I_{4} & =\frac{1}{2}\sum_{n=0}^{\infty }\frac{{\left(\frac{\left(x-N\mu \right)}{N\sigma^{2}}\right)}^{n}}{n!}\\ \; & \times \int_{0}^{\infty }\;t^{\phi_{0}+n}H_{1,2}^{2,0}\left[\frac{\mu_{0}\;t^{\alpha_{0}}}{{\left(S_{0}H\hat{h_{0}}\right)}^{\alpha_{0}}}|\;\begin{array}{c} \left(1,1\right)\\ \left(k-\frac{\phi_{0}}{\alpha_{0}},1\right),\left(0,1\right) \end{array}\;\right]\\ \; & \times H_{0,1}^{1,0}\left[\sqrt{\frac{1}{2N\sigma^{2}}}t|\begin{array}{c} ¯\\ \left(0,\frac{1}{2}\right) \end{array}\right]\;dt. \end{array}$$

Let $\sqrt{\frac{1}{2N\sigma^{2}}}\;t$ = *s*, then $I_{4}$ would become

(A31) $$\begin{array}{cc} I_{4} & =\frac{1}{2}{\left(2N\sigma^{2}\right)}^{\frac{\phi_{0}+1}{2}}\sum_{n=0}^{\infty }\frac{{\left(\sqrt{\frac{2}{N\sigma^{2}}}\left(x-N\mu \right)\right)}^{n}}{n!}\int_{0}^{\infty }s^{\phi_{0}+n}\\ \; & \times H_{1,1}^{2,0}\left[\mu_{0}\;{\left(\frac{\sqrt{2N\sigma^{2}}}{S_{0}\;\hat{h_{0}}\;H}\right)}^{\alpha_{0}}s^{\alpha_{0}}|\begin{array}{c} \left(1,1\right)\\ \left(k-\frac{\phi_{0}}{\alpha_{0}},1\right),\left(0,1\right) \end{array}\right]\\ \; & \times H_{0,1}^{1,0}\left[s|\begin{array}{c} ¯\\ \left(0,\frac{1}{2}\right) \end{array}\right]\;ds. \end{array}$$

$I_{4}$ can be solved using [17] Equation (2.6.8) as

(A32) $$\begin{array}{cc} I_{4} & =\frac{1}{2}{\left(2N\sigma^{2}\right)}^{\frac{\phi_{0}+1}{2}}\sum_{n=0}^{\infty }\frac{{\left(\sqrt{\frac{2}{N\sigma^{2}}}\left(x-N\mu \right)\right)}^{n}}{n!}\\ \; & \times H_{2,2}^{2,1}\left[\mu_{0}\;{\left(\frac{\sqrt{2N\sigma^{2}}}{S_{0}\;\hat{h_{0}}\;H}\right)}^{\alpha_{0}}|\begin{array}{c} \left(\frac{1}{2}\left(1-\phi_{0}-n\right),\frac{\alpha_{0}}{2}\right),\left(1,1\right)\\ \left(k-\frac{\phi_{0}}{\alpha_{0}},1\right),\left(0,1\right) \end{array}\right] \end{array}$$

Substituting $I_{3}$ and $I_{4}$ in (A27), we obtain the desired result and hence the proof is complete. □

## References

[1] Saad W., Bennis M., Chen M. (2020). A Vision of 6G Wireless Systems: Applications, Trends, Technologies, and Open Research Problems. IEEE Netw..

[2] Liu R., Wu Q., Di Renzo M., Yuan Y. (2022). A Path to Smart Radio Environments: An Industrial Viewpoint on Reconfigurable Intelligent Surfaces. IEEE Wireless Commun..

[3] Ghavami H., Akhbari B. (2023). Secrecy performance analysis of IRS-NOMA systems. J. Wirel. Commun. Netw..

[4] Papasotiriou E.N., Boulogeorgos A.A.A., Haneda K., de Guzman M.F., Alexiou A. (2021). An experimentally validated fading model for THz wireless systems. Sci. Rep..

[5] Kudathanthirige D., Gunasinghe D., Amarasuriya G. Performance Analysis of Intelligent Reflective Surfaces for Wireless Communication. Proceedings of the IEEE ICC.

[6] Tao Q., Wang J., Zhong C. (2020). Performance Analysis of Intelligent Reflecting Surface Aided Communication Systems. IEEE Commun. Lett..

[7] Cheng Y., Li K.H., Liu Y., Teh K.C. Outage Performance of Downlink IRS-Assisted NOMA Systems. Proceedings of the IEEE Globecom.

[8] Bhardwaj P., Zafaruddin S.M. (2021). Performance of Dual-Hop Relaying for THz-RF Wireless Link Over Asymmetrical *α*-*μ* Fading. IEEE Trans. Veh. Technol..

[9] Li S., Yang L. (2022). Performance Analysis of Dual-Hop THz Transmission Systems Over *α*-*μ* Fading Channels with Pointing Errors. IEEE Internet Things J..

[10] Chapala V.K., Zafaruddin S.M. (2021). Exact Analysis of RIS-Aided THz Wireless Systems Over *α*-*μ* Fading with Pointing Errors. IEEE Commun. Lett..

[11] Li S.J., Han B.W., Li Z.Y., Liu X.B., Huang G.S., Li R.Q., Cao X.Y. (2022). Transmissive coding metasurface with dual-circularly polarized multi-beam. Opt. Express.

[12] Li S.J., Li Z.Y., Liu X.B., He C., Huang G.S., Li R.Q., Cao X.Y. (2023). Transmissive Digital Coding Metasurfaces for Polarization-Dependent Dual-Mode Quad Orbital Angular Momentum Beams. ACS Appl. Mater. Interfaces.

[13] Boulogeorgos A.A.A., Papasotiriou E.N., Alexiou A. (2019). Analytical Performance Assessment of THz Wireless Systems. IEEE Access.

[14] Yacoub M.D. (2007). The *α*-*μ* Distribution: A physical fading model for the Stacy distribution. IEEE Trans. Veh. Technol..

[15] Gradshteyn I.S., Ryzhik I.M. (2007). Table of Integrals, Series and Products.

[16] Boulogeorgos A.A., Alexiou A. (2020). Error Analysis of Mixed THz-RF Wireless Systems. IEEE Commun. Lett..

[17] Mathai A.M., Saxena R.K. (1978). The H-Function with Applications in Statistics and Other Disciplines.

[18] Bodenschatz C.D. (1992). Finding an H-Function Distribution for the Sum of Independent H-Function Variates. Ph.D. Thesis.

